# A pan-cancer analysis confirms PTPN11’s potential as a prognostic and immunological biomarker

**DOI:** 10.18632/aging.204171

**Published:** 2022-07-08

**Authors:** Yapeng Cao, Haixia Duan, Ailing Su, Liran Xu, Baochang Lai

**Affiliations:** 1 Cardiovascular Research Center, School of Basic Medical Sciences, Xi’an Jiaotong University Health Science Center, Key Laboratory of Environment and Genes Related to Diseases, Xi’an Jiaotong University, Ministry of Education, Xi’an, Shaanxi 710061, China; 2 Department of Reproduction Gynecology, Northwest Women and Children’s Hospital, Xi’an, Shaanxi 710061, China

**Keywords:** PTPN11, pan-cancer, prognosis, phosphorylation, immune infiltration

## Abstract

Protein tyrosine phosphatase, non-receptor type 11 (PTPN11) is a multifunctional tyrosine phosphatase and has a significant part in many types of tumors. As of yet, neither the expression profile of PTPN11 nor its significance in pan-cancer diagnosis has been clarified. With the assistance of The Cancer Genome Atlas (TCGA) and Gene Expression Omnibus (GEO), we have comprehensively mapped the expression profiles, prognostic significance, genetic alteration, phosphorylation status, infiltration of immune cells, and functional properties of PTPN11 in 33 human tumors. There was an inconsistent expression of *PTPN11* in different tumors, and the alteration of *PTPN11* expression predicted the survival outcomes of cancer patients. A significant association was found between the genetic alteration levels of *PTPN11* and some tumor predictions. Besides, the reduced PTPN11 phosphorylation levels were observed in breast cancer, clear cell RCC, head and neck carcinoma, and lung adenocarcinoma (LUAD). Furthermore, there was a significant association between *PTPN11* expression and infiltration of cancer-associated fibroblasts and endothelial cells, along with tumor mutation burden, microsatellite instability, mismatch repair genes, and immunoregulators. Finally, pathway enrichment analysis demonstrated that PTPN11-associated terms and pathways were involved in malignancy. Taken together, PTPN11 may become a new biomarker and target for cancer therapy.

## INTRODUCTION

Due to its leading cause of death and morbidity, cancer has made it one of the greatest risks to public health worldwide. Cancer development is a complex process influenced by the origin of cells, the tumor location, and genomic changes as well as the inherited and acquired molecular and cellular alterations [1–3]. Although many drugs and therapies have been developed to treat cancer, people are still not satisfied with the current therapies for cancer by reason of serious drug side effects, drug tolerance, expensive treatment costs, and missed targets. Therefore, there is still a great need to shed light on the accurate molecular mechanisms of oncogenesis and explore better prognostic markers for cancer prognosis. As whole-genome sequencing technology has rapidly advanced, many public cancer datasets, such as TCGA and GEO, have been established over the last few years [4, 5]. Thanks to the open access of these public databases, we can find out the clinical outlook of genes of interest via a pan-cancer analysis, as well as commonalities and differences in human tumors [6].

PTPN11, which is also referred to as SHP2, is the first tyrosine phosphatase to be identified as oncogenic [7], and is found in many tissues and cells. PTPN11 includes two tandem C-SH2 and N-SH2 domains, as well as two tyrosine phosphorylation sites (Tyr542 and Tyr580) at the C-terminus, in addition to the protein tyrosine phosphatase catalytic domain (PTP domain) that is located somewhere at C-terminus [8]. According to the previous reports, a variety of intracellular events are regulated by PTPN11, including mitogenic activation, tumor cell proliferation, invasion, metastasis, apoptosis, senescence, and differentiation of multiple cell types [9–11]. And the germline gain of function mutations in the *PTPN11* gene is the cause of Noonan syndrome (NS) and juvenile myelomonocytic leukemia (JMML) via triggering Ras/Erk signaling pathway [12, 13]. Additionally, PTPN11 has been regarded as a vital oncogene that has been intensively studied in some cancers, such as breast cancer [14], and melanoma [15]. However, PTPN11 shows a tumor-suppressive function in liver cancer [16], suggesting that PTPN11 plays different biological roles in different tumor cells. Although PTPN11 has been studied separately in several tumors, pan-cancer evidence has yet to be established to elucidate the potential impact of PTPN11 in divergent malignancies in the previous researches.

In our present research, therefore, we evaluated the pan-cancer properties of PTPN11 by means of the TCGA project, GEO databases, the Clinical Proteomic Tumor Analysis Consortium (CPTAC), and the Human Protein Atlas (HPA) cohort in order to establish the link between PTPN11 expression and prognosis. Also, the correlation of PTPN11 expression with genetic mutation, protein phosphorylation, immune cell infiltration, tumor mutation burden (TMB), microsatellite instability (MSI), and mismatch repair genes (MMRs), as well as the underlying cellular pathway was identified, which suggests that PTPN11 may function as a potentially valuable marker for cancer treatment.

## RESULTS

### Expression of *PTPN11* differently in human cancers

**Figure 1:**
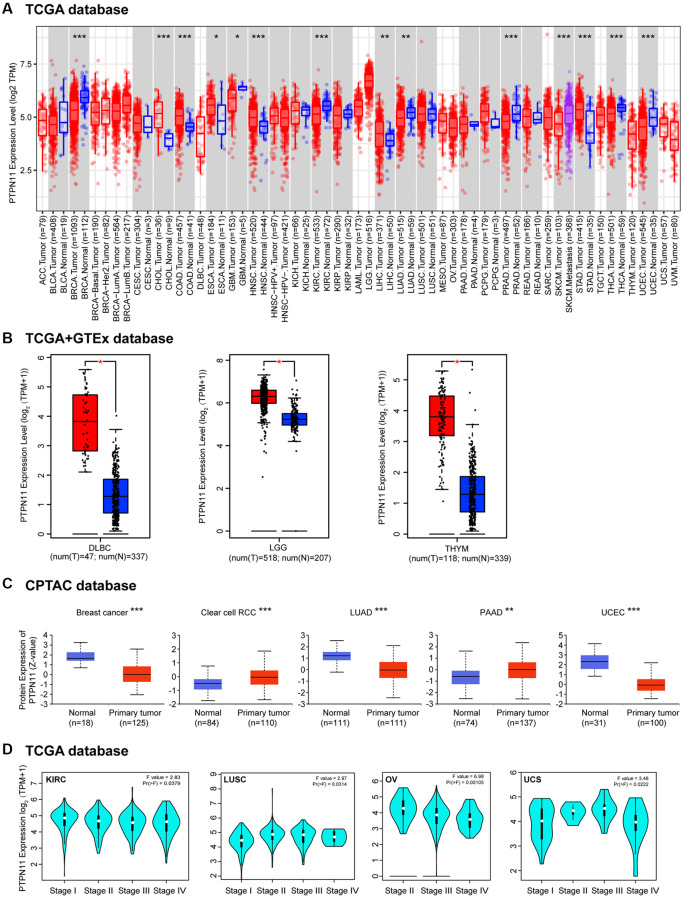
**The differential expression of *PTPN11* gene in various tumors and pathological phases.** (**A**) The expression of *PTPN11* gene in various cancers or specific subcategories of tumor. ^*^p < 0.05; ^**^p < 0.01; ^***^p < 0.001. (**B**) The expression of *PTPN11* in DLBC, LGG, and THYM in the TCGA project was compared with the comparable healthy tissues in the GTEx dataset. ^*^p < 0.05. (**C**) On the basis of the CPTAC database, the expression levels of PTPN11 total protein in healthy and primary tissues were evaluated for breast cancer, clear cell RCC, LUAD, PAAD, and UCEC. ^**^p < 0.01; ^***^p < 0.001. (**D**) According to TCGA data, the expression levels of *PTPN11* were analyzed by the primary pathological stages (stages I, II, III, and IV) of KIRC, LUSC, OV, and UCS. For the log scale, Log2 (TPM + 1) was applied.

As part of our study of the role of PTPN11 in tumors, we examined *PTPN11* levels of expression throughout the TCGA database. We discovered that *PTPN11* was substantially expressed in cholangiocarcinoma (CHOL), colon adenocarcinoma (COAD), esophageal carcinoma (ESCA), head and neck squamous cell carcinoma (HNSC), liver hepatocellular carcinoma (LIHC), and stomach adenocarcinoma (STAD) using the TIMER2 algorithm (Figure 1A). However, expression of *PTPN11* was found to be relatively lesser in breast invasive carcinoma (BRCA), glioblastoma multiforme (GBM), kidney renal clear cell carcinoma (KIRC), LUAD, prostate adenocarcinoma (PRAD), thyroid carcinoma (THCA), and uterine corpus endometrial carcinoma (UCEC) compared to that of relating healthy tissues (Figure 1A). Additionally, we applied the GTEx dataset to further corroborate the expression profiles of *PTPN11* in several other cancers, that lacked healthy tissues matched in TIMER2 dataset. The results suggested expression levels of *PTPN11* in lymphoid neoplasm diffuse large B-cell lymphoma (DLBC), brain lower-grade glioma (LGG), and thymoma (THYM) were significantly greater than that of corresponding normal tissues (Figure 1B). Differential expression of *PTPN11* in divergent tumors indicated that PTPN11 plays different roles in various tumors.

Furthermore, an analysis of the total protein levels of PTPN11 in various tumors was performed employing the CPTAC database. Figure 1C demonstrates that the protein expression of PTPN11 in renal clear cell carcinoma (RCC) and pancreatic adenocarcinoma (PAAD) was substantially elevated. Yet, we found PTPN11 protein levels were significantly downregulated in LUAD, breast cancer, and UCEC (Figure 1C).

Then “Pathologic Stage Plot” module in GEPIA2 was utilized in order to examine how *PTPN11* expression correlated with the pathologic phase of cancers. Strong correlations were found in KIRC, lung squamous cell carcinoma (LUSC), ovarian serous cystadenocarcinoma (OV), and uterine carcinosarcoma (UCS) (Figure 1D).

### The prognostic value of PTPN11 in human pan-cancer

**Figure 2:**
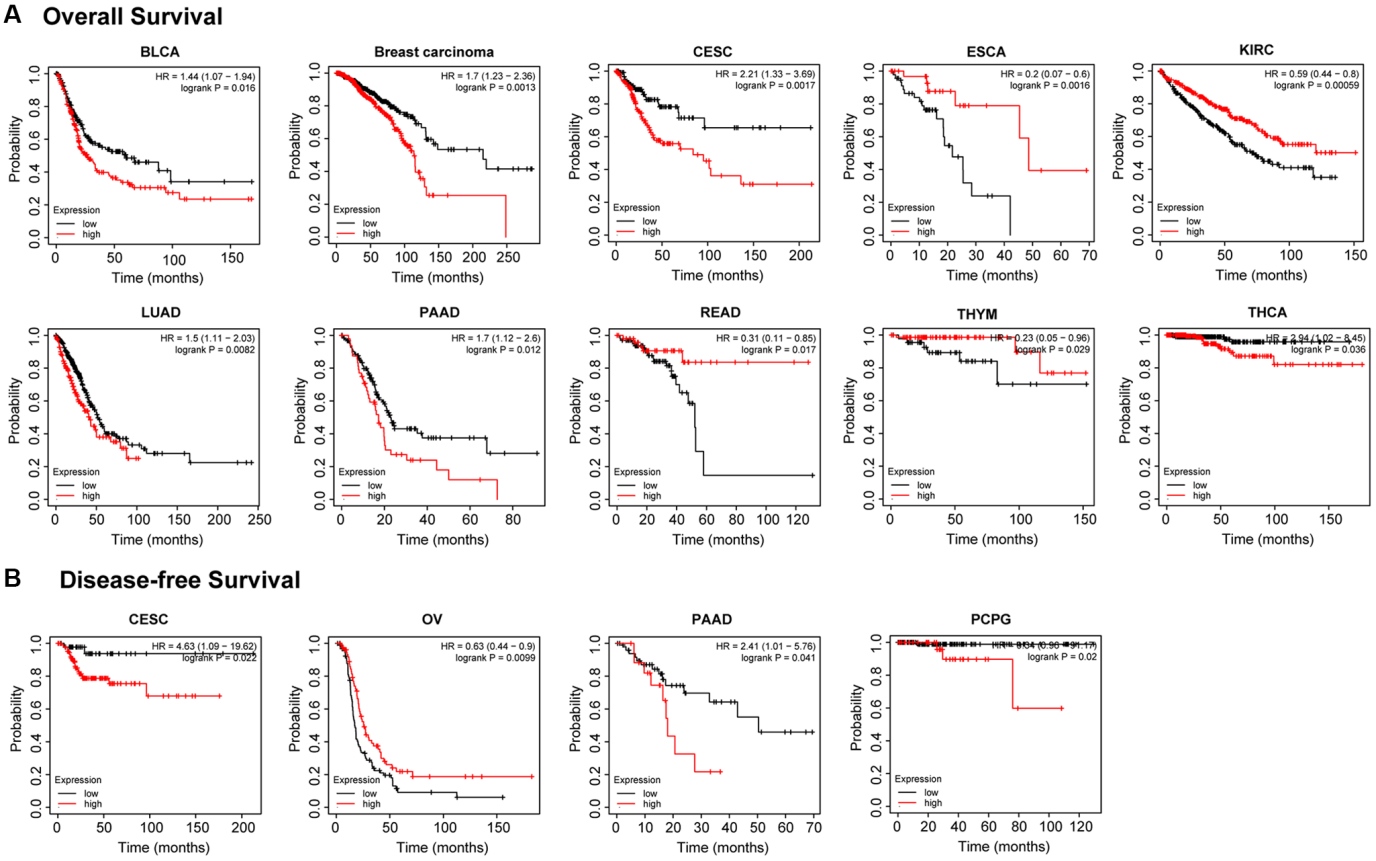
**Kaplan-Meier analysis of the relationship between *PTPN11* expression and survival features of tumors in TCGA.** (**A**) The examination of the link between *PTPN11* expression and OS for BLCA, BRCA, CESC, ESCA, KIRC, LUAD, PAAD, READ, THYM, and THCA. (**B**) The investigation of the association between *PTPN11* expression and DFS in CESC, OV, PAAD, and PCPG.

We conducted a survival correlation study for every malignancy using a Kaplan-Meier plotter in order to get more insight into the association between *PTPN11* expression and prognostic value across diverse tumor types. As can be seen in Figure 2A, individuals who had elevated expression levels of *PTPN11* were significantly linked to poor overall survival (OS) in the following cancers: bladder carcinoma (BLCA) (*p* = 0.016), breast cancer (*p* = 0.0013), cervical squamous cell carcinoma and endocervical adenocarcinoma (CESC) (*p* = 0.0017), LUAD (*p* = 0.0082), PAAD (*p* = 0.012), and thyroid carcinoma (THCA) (*p* = 0.036). However, higher expression of *PTPN11* was related to longer OS in cases with ESCA (*p* = 0.0016), KIRC (*p* < 0.001), rectum adenocarcinoma (READ) (*p* = 0.017), and THYM (*p* = 0.029).

In terms of the disease-free survival (DFS) as illustrated in Figure 2B, highly expressed *PTPN11* was significantly correlated with poor DFS in CESC (*p* = 0.022), PAAD (*p* = 0.041), and pheochromocytoma and paraganglioma (PCPG) (*p* = 0.02), while low expression of *PTPN11* has poor DFS in OV (*p* = 0.0099).

### Analysis of *PTPN11* genetic mutations in human pan-cancer

**Figure 3:**
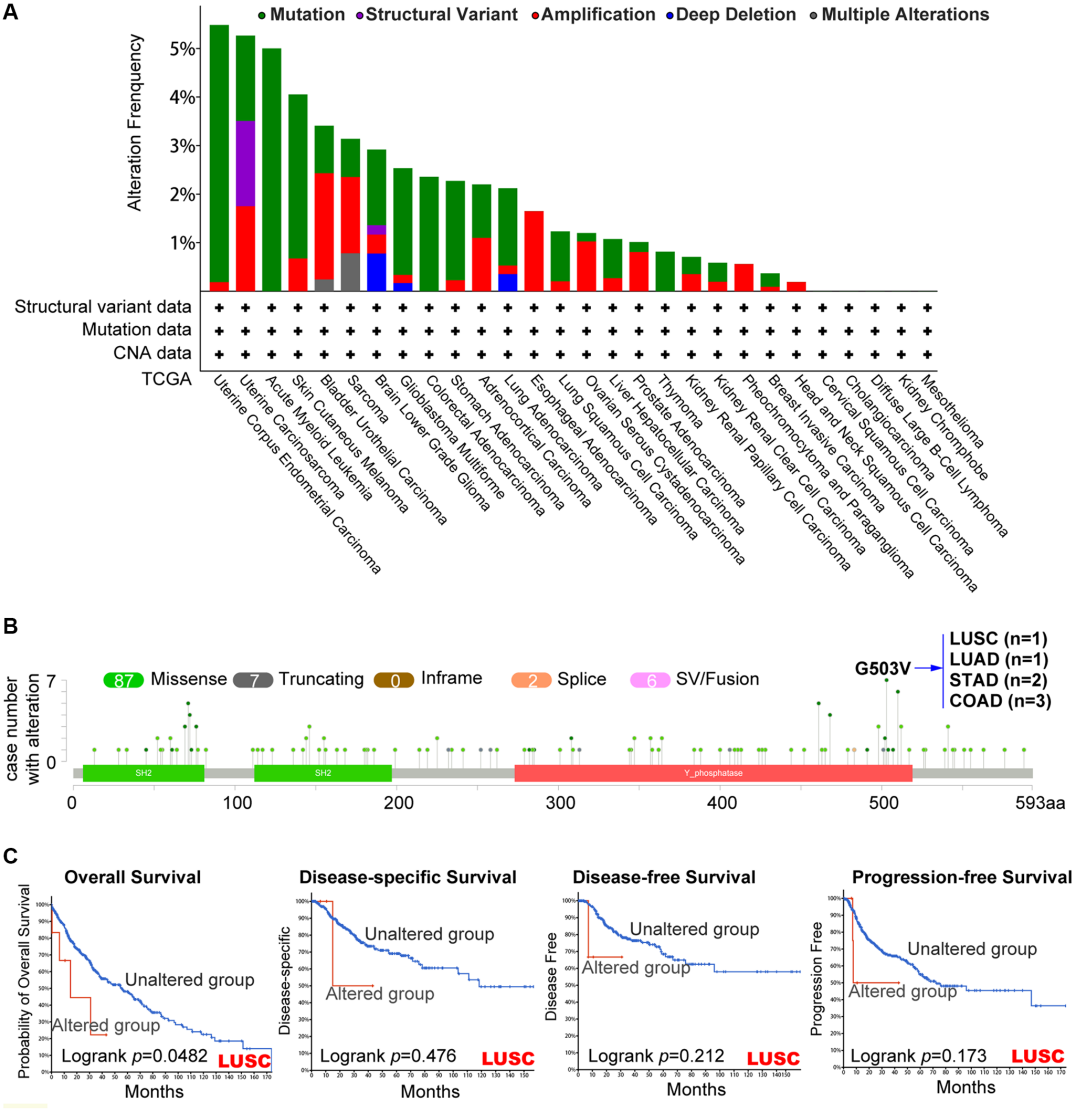
**Mutation’s characteristic of *PTPN11* in a variety of TCGA cancers.** The cBioPortal application displays the frequency of *PTPN11* mutations with mutation type (**A**) and mutation locations (**B**) in TCGA cancers. (**C**) The cBioPortal tool was utilized to evaluate the possible relation between *PTPN11* mutation state and overall, disease-specific, disease-free, and progression-free survival of LUSC patients.

Using the cBioPortal tool, we evaluated the genetic modification state of *PTPN11* in distinct cancers from TCGA dataset. Based on Figure 3A, mutations were the most frequent alteration of *PTPN11*, mainly in uterine tumors (>5%). The type, locations, and case numbers of *PTPN11* genetic change were shown to us in Figure 3B. It was a missense mutation that was the most prevalent genetic alteration for *PTPN11* among them. G503V alteration in the domain of Y_phosphatase of *PTPN11* was found in one case of LUSC, one case of LUAD, two cases of STAD, and three cases of COAD. Additionally, we assessed whether genetic alterations of *PTPN11* was associated with tumor patient survival prospects. The results confirmed that LUSC cases with altered *PTPN11* genetic alteration have poorer prognosis in OS (*p* = 0.0482) compared with the unaltered cases (Figure 3C), however, disease-specific survival (DSS) (*p* = 0.476), DFS (*p* = 0.212), and progression-free survival (PFS) (*p* = 0.173) were not substantially different between these two groups (Figure 3C).

### The protein expression analysis of PTPN11 in human pan-cancer

**Figure 4:**
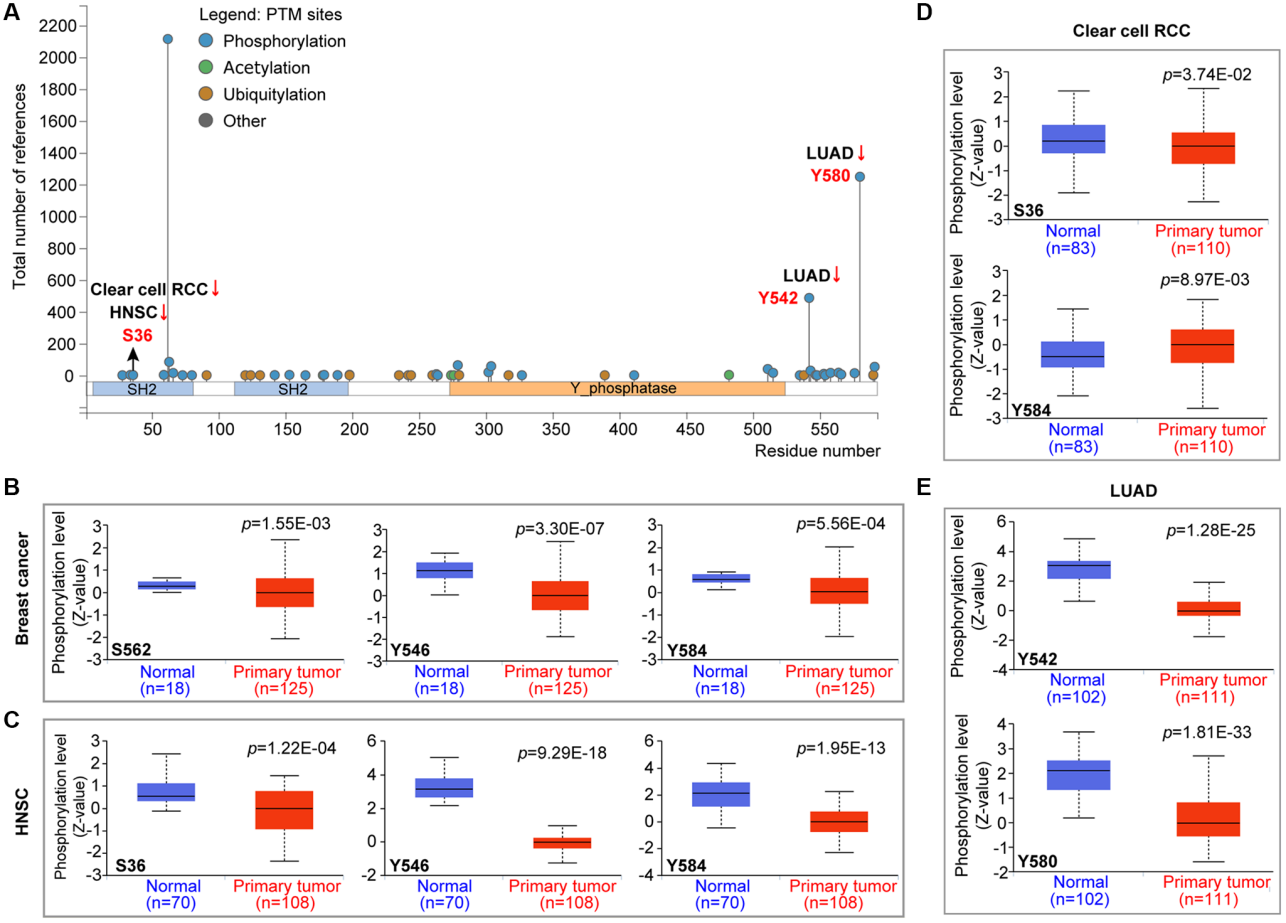
**Phosphorylation analysis of PTPN11 protein in various cancers according to the CPTAC database.** It was retrieved from the UALCAN in order to compare the phosphorylation levels of PTPN11 (NP 002825.3, S36, S562, Y546, Y584, Y542, and Y580) in several malignant tissues to that of normal tissues. (**A**) The phosphorylation sites of the PTPN11 protein are depicted in the diagram. The box plots are shown for several malignancies, such as (**B**) breast cancer, (**C**) HNSC, (**D**) clear cell RCC, and (**E**) LUAD.

PTPN11 was the first identified carcinogenic phosphatase. We examined the changes of PTPN11 phosphorylation levels among cancer tissues and corresponding healthy tissues using the CPTAC database. According to Figure 4A, the phosphorylation level of S36 of PTPN11 was remarkably reduced in malignant tissues of HNSC and clear cell RCC (Figure 4C, 4D). The phosphorylation levels of Y546 and Y584 of PTPN11 were lower in tumor tissues of breast cancer and HNSC (Figure 4B, 4C). The phosphorylation degree of S562 of PTPN11 was substantially decreased in tumor tissues of breast cancer (Figure 4B). Also, the phosphorylation levels of Y542 and Y580 of PTPN11 were remarkably lower in tumor tissues of LUAD (Figure 4A and 4E). However, higher protein phosphorylation levels of Y584 of PTPN11 was noted in tumor tissues of clear cell RCC (Figure 4D).

**Figure 5:**
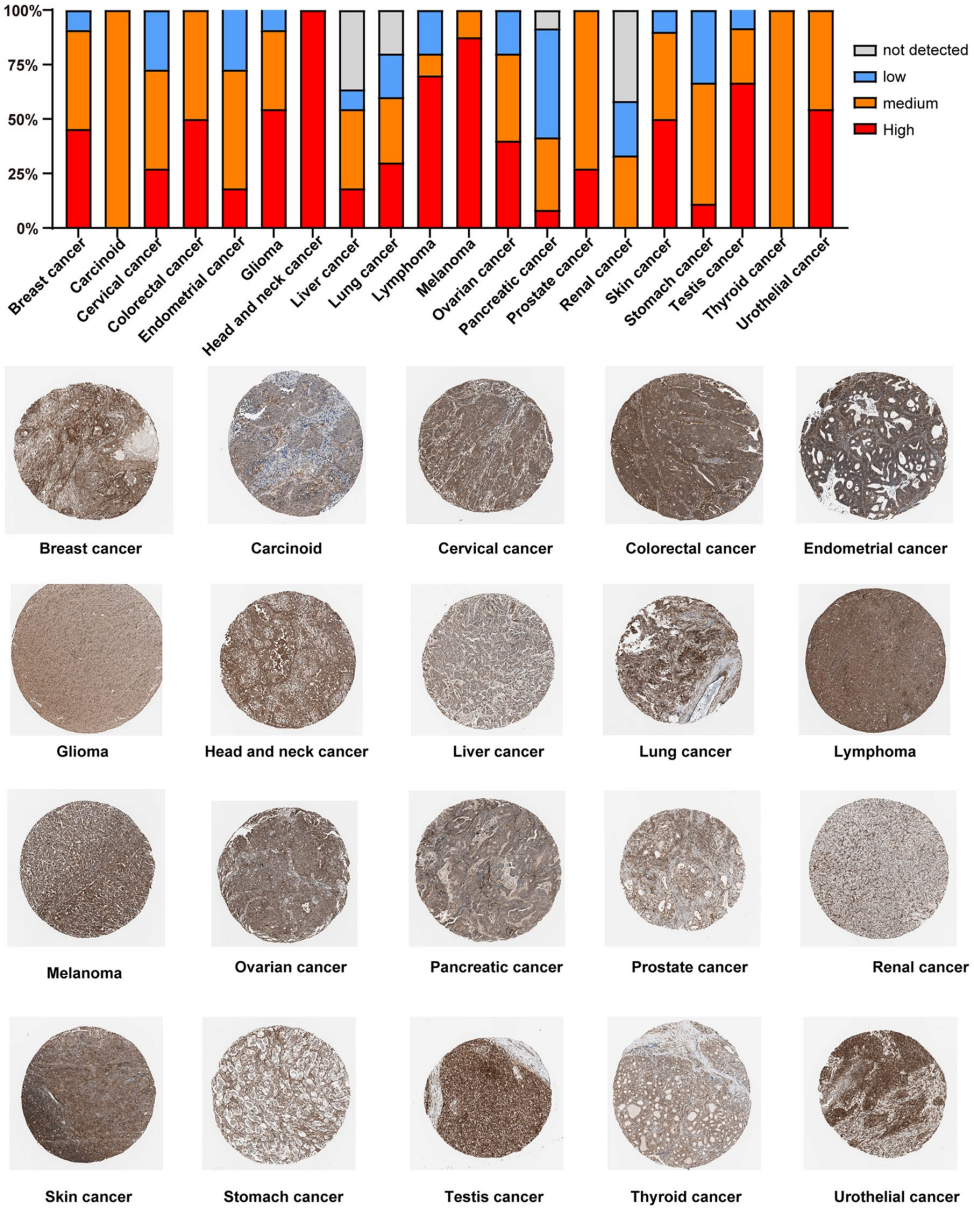
Immunohistochemical staining analysis of the PTPN11 protein in various TCGA tumor tissues via HPA database.

Furthermore, we detected the PTPN11 protein expression levels in different cancers by the HPA cohort. We found that high PTPN11 expression levels were obtained in most types of cancer, including HNSC, melanoma, lymphoma, testis cancer, GBM, urothelial cancer, COAD, SKCM, BRCA, OV, lung cancer, CESC, PRAD, endometrial cancer, liver cancer, STAD, and PAAD (Figure 5).

### Investigation of immune cell infiltration of PTPN11 in human pan-cancer

**Figure 6:**
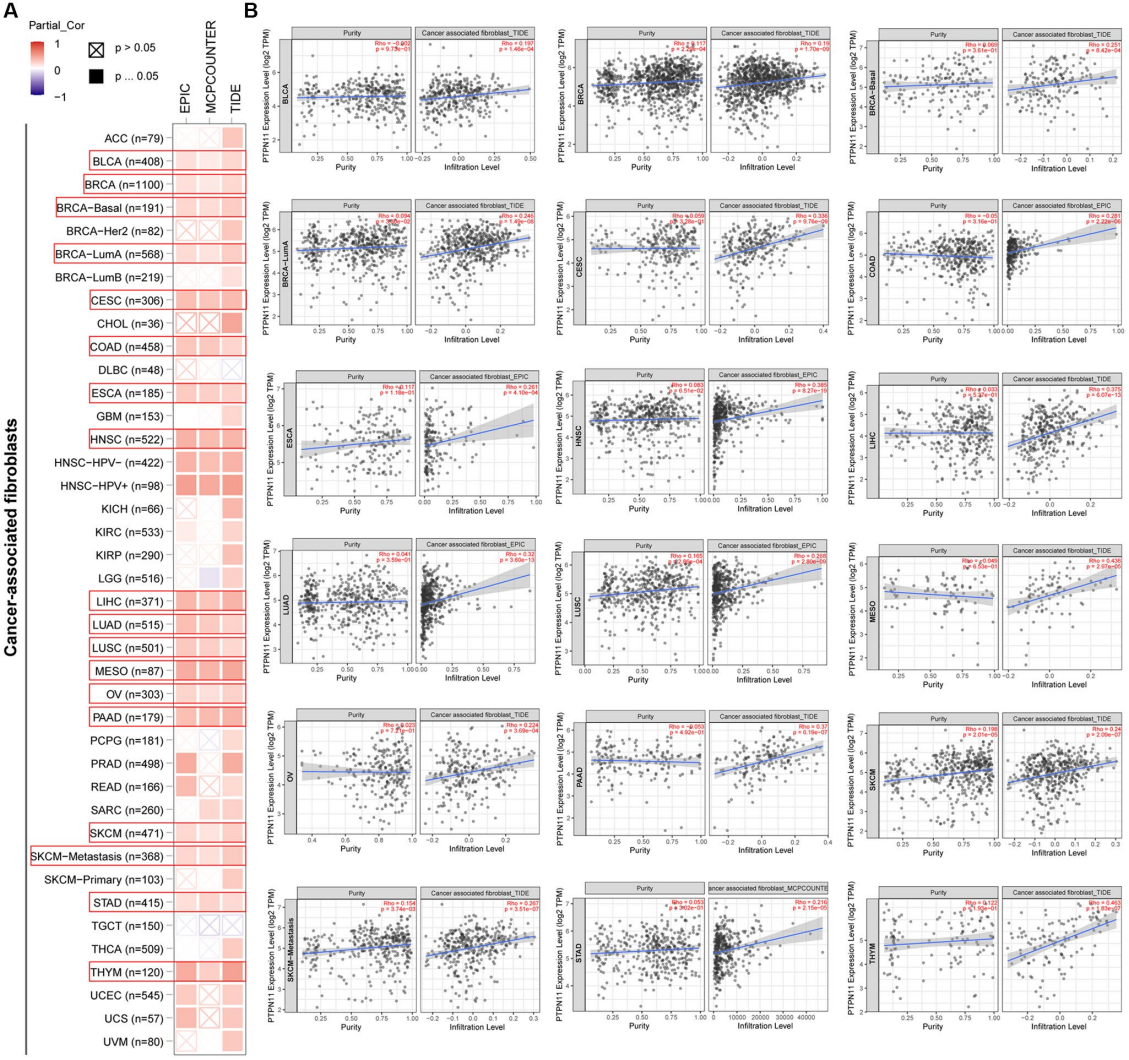
**Analysis of the relationship between *PTPN11* expression and immune infiltration of cancer-associated fibroblasts.** (**A**) Using the EPIC, MCPCOUNTEER, and TIDE algorithms, the connection between *PTPN11* expression and the amount of cancer-associated fibroblast infiltration was assessed. (**B**) The correlation between *PTPN11* expression and cancer-associated fibroblast infiltration in BLCA, BRCA, CESC, COAD, ESCA, HNSC, LIHC, LUAD, LUSC, MESO, OV, PAAD, SKCM, STAD, and THYM.

**Figure 7:**
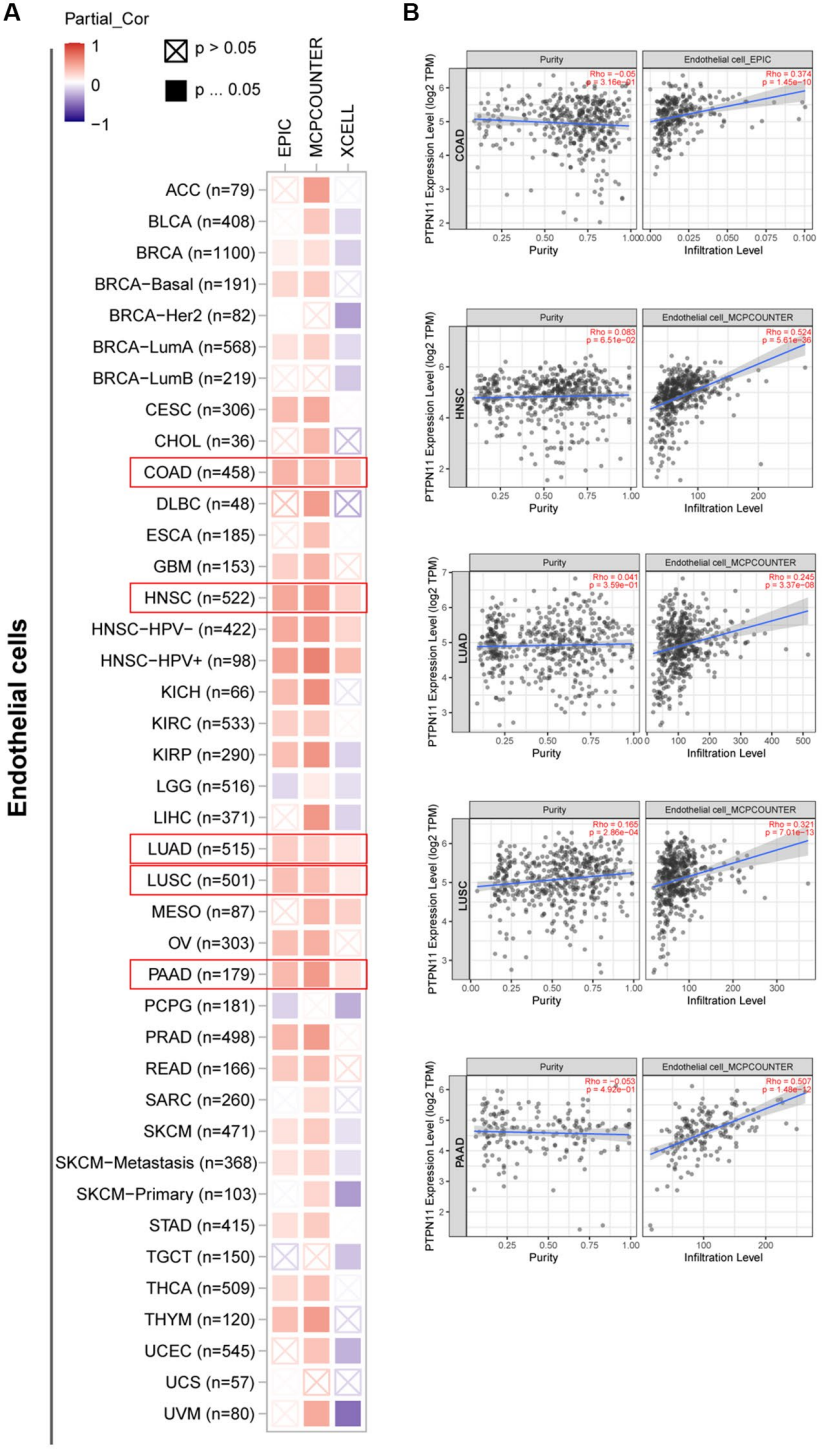
**Investigation of the association between *PTPN11* expression and immune infiltration of endothelial cells.** (**A**) Using the EPIC, MCPCOUNTEER, and XCELL algorithms, a link was found between *PTPN11* expression and the amount of endothelial cell infiltration. (**B**) The relationship between *PTPN11* expression and endothelial cell infiltration levels in COAD, HNSC, LUAD, LUSC, and PAAD.

Earlier research has confirmed that tumor microenvironment (TME) can contribute to tumor development and foster the evasion of the immune system by tumor cells [17–19]. Therefore, treatment response and clinical outcome of cancer are highly influenced by TME. Cancer cells, infiltrating immune cells, and stromal cells make up the TME. Infiltrating immune cells are regarded as the dominant elements of TME, and exert a substantial influence on tumorigenesis, progression, and metastasis [20]. It has been demonstrated that cancer-associated fibroblasts and endothelial cells contribute to the progression of tumor in the TME [21, 22]. Consequently, we examined the relationship between *PTPN11* expression and tumor-infiltrating immune cells in fibroblasts and endothelial cells related to cancer. As presented in Figure 6A and 6B, a positively significant association existed between *PTPN11* expression and the infiltration abundance of cancer-related fibroblasts in TCGA tumors comprising BLCA, BRCA, CESC, COAD, ESCA, HNSC, LIHC, LUAD, LUSC, MESO, OV, PAAD, SKCM, STAD, and THYM. In addition, Figure 7A and 7B demonstrate a statistically significant association between *PTPN11* expression and immune infiltration of endothelial cells in COAD, HNSC, LUAD, LUSC, and PAAD. Collectively, these results confirmed that PTPN11 was crucial for regulating the TME.

### Correlation analysis of *PTPN11* expression with TMB, MSI, and MMRs in human pan-cancer

**Figure 8:**
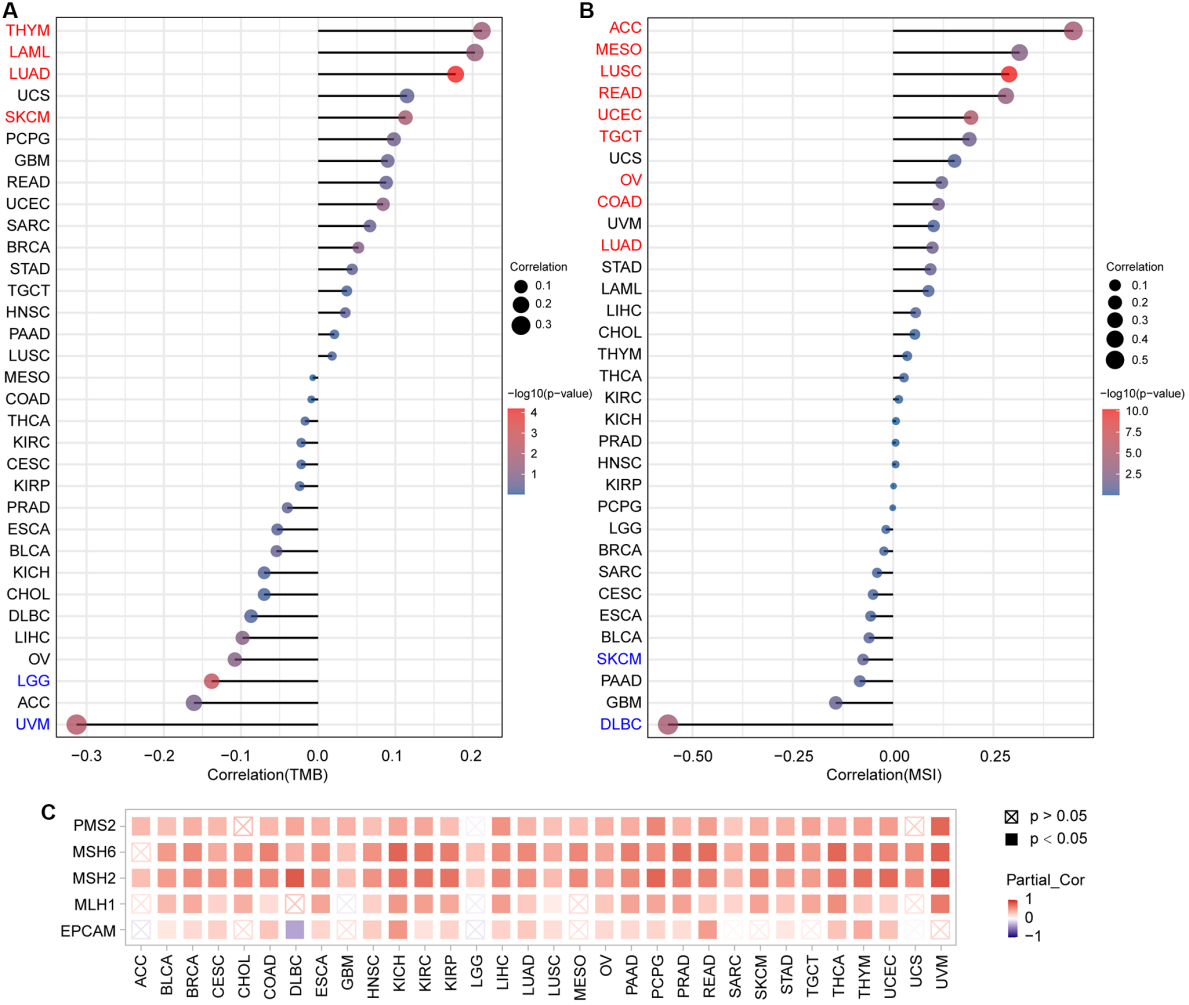
**Analysis of the relationship between *PTPN11* expression and TMB, MSI, and MMRs in human pan-cancer.** (**A**) A stick chart depicts the association between *PTPN11* expression and TMB in various malignancies. (**B**) A stick chart depicts the link between *PTPN11* expression and MSI in various cancers. (**C**) Correlation between the expression of *PTPN11* and MMRs.

TMB is a potential new immunotherapy response marker. Additionally, MSI acts as a biomarker of immune-checkpoint blockers that is directly linked to the progression of most tumors [23, 24]. As presented in Figure 8A and Supplementary Figure 1A, *PTPN11* expression was correlated positively with TMB in THYM, LAML, LUAD, and SKCM; and correlated negatively with TMB in LGG and UVM. Further analysis of *PTPN11* expression revealed positive correlations with MSI in nine types of cancer, including ACC, MESO, LUSC, READ, UCEC, TGCT, OV, COAD, and LUAD; and negative correlations with MSI in SKCM and DLBC (Figure 8B and Supplementary Figure 1B).

Maintaining the genome stability relies heavily on the correct replication of the genome. MMRs can maintain the genome stability against spontaneous DNA damage. In view of this, we examined whether or not expression of *PTPN11* was associated with MMRs. According to our findings, *PTPN11* expression was related in a positive way to the five MMR genes (*EPCAM, MLH1, MSH2, MSH6*, and *PMS2*) in 21 cancers, including BLCA, BRCA, CESC, COAD, ESCA, HNSC, KICH, KIRC, KIRP, LIHC, LUAD, LUSC, OV, PAAD, PCPG, PRAD, READ, STAD, THCA, THYM, and UCEC (Figure 8C).

### Analysis of the relationship between *PTPN11* expression and immunoregulators in human pan-cancer

**Figure 9:**
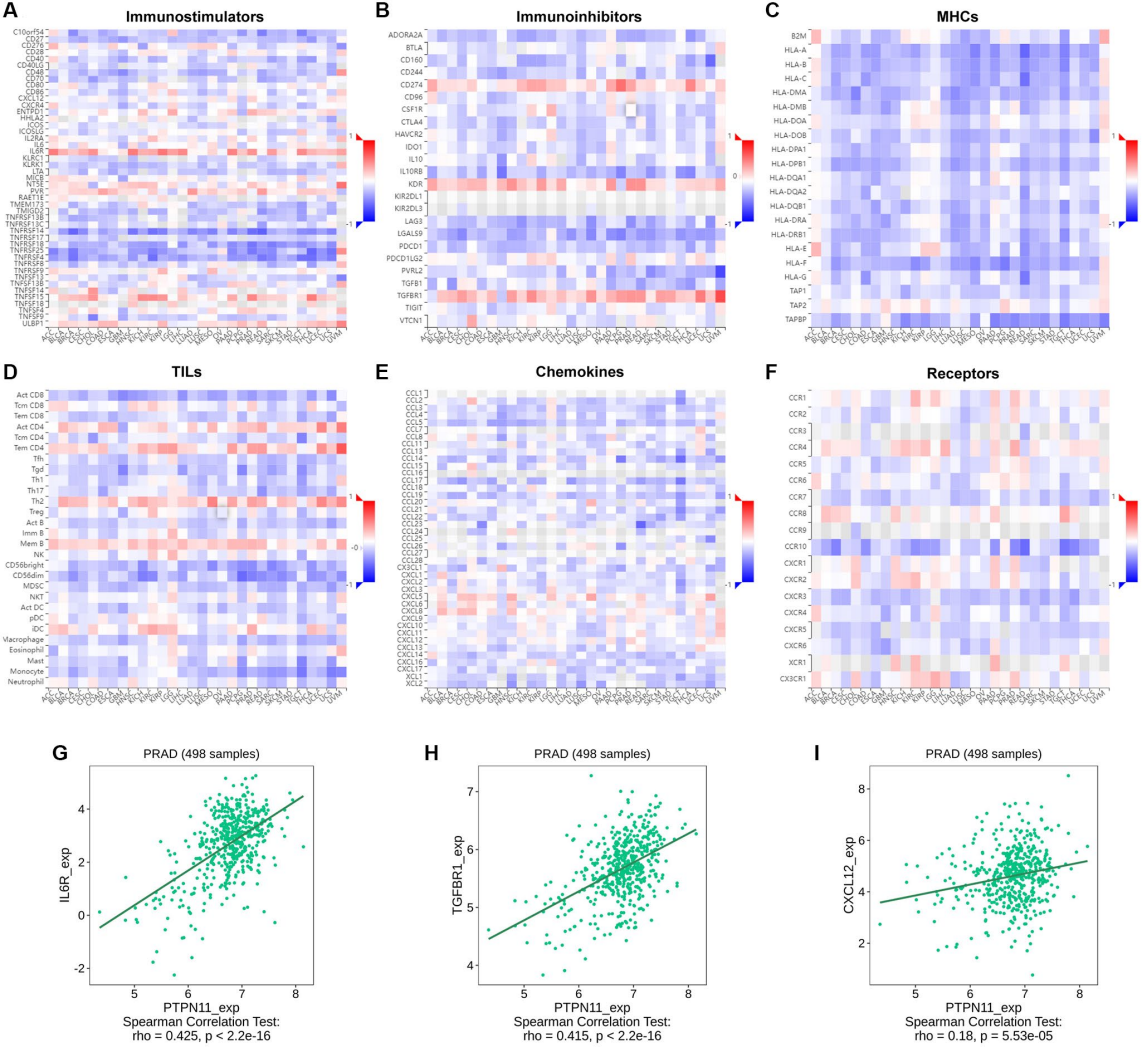
**Relationship between the expression of *PTPN11* and immunoregulators.** (**A**–**F**) The link between *PTPN11* expression and immunostimulators, immunoinhibitors, MHC molecules, TILs, chemokines, and receptors in diverse human malignancies. (**G**–**I**) The association between *PTPN11* expression and IL6R, TGFBR1, and CXCL12 expression in PRAD.

Further, a gene co-expression study was performed to investigate the relationships between the expression of *PTPN11* and immune-associated genes and tumor-infiltrating lymphocytes (TILs) across multiple kinds of human tumors. MHC, immunological stimulation, immunosuppression, chemokine, and chemokine receptor proteins were encoded by the immune-related genes examined. Our results suggested that nearly all the immune-related genes were significantly related to *PTPN11* and most were negatively related to *PTPN11* across most cancers (Figure 9A–9F).

In PRAD, we found *PTPN11* was remarkably positively related to several immunomodulators, including immunostimulator IL6R expression (spearman correlation of 0.425), immunoinhibitor TGFBR1 expression (spearman correlation of 0.415), and CXCL12 expression (spearman correlation of 0.18) (Figure 9G–9I), which indicated PTPN11 might regulate the immunomodulators IL6R, TGFBR1, and CXCL12 in PRAD. Therefore, the above results confirmed that PTPN11 might be responsible for regulating the immune cell function in TME.

### Functional enrichment analysis of PTPN11 in human pan-cancer

**Figure 10:**
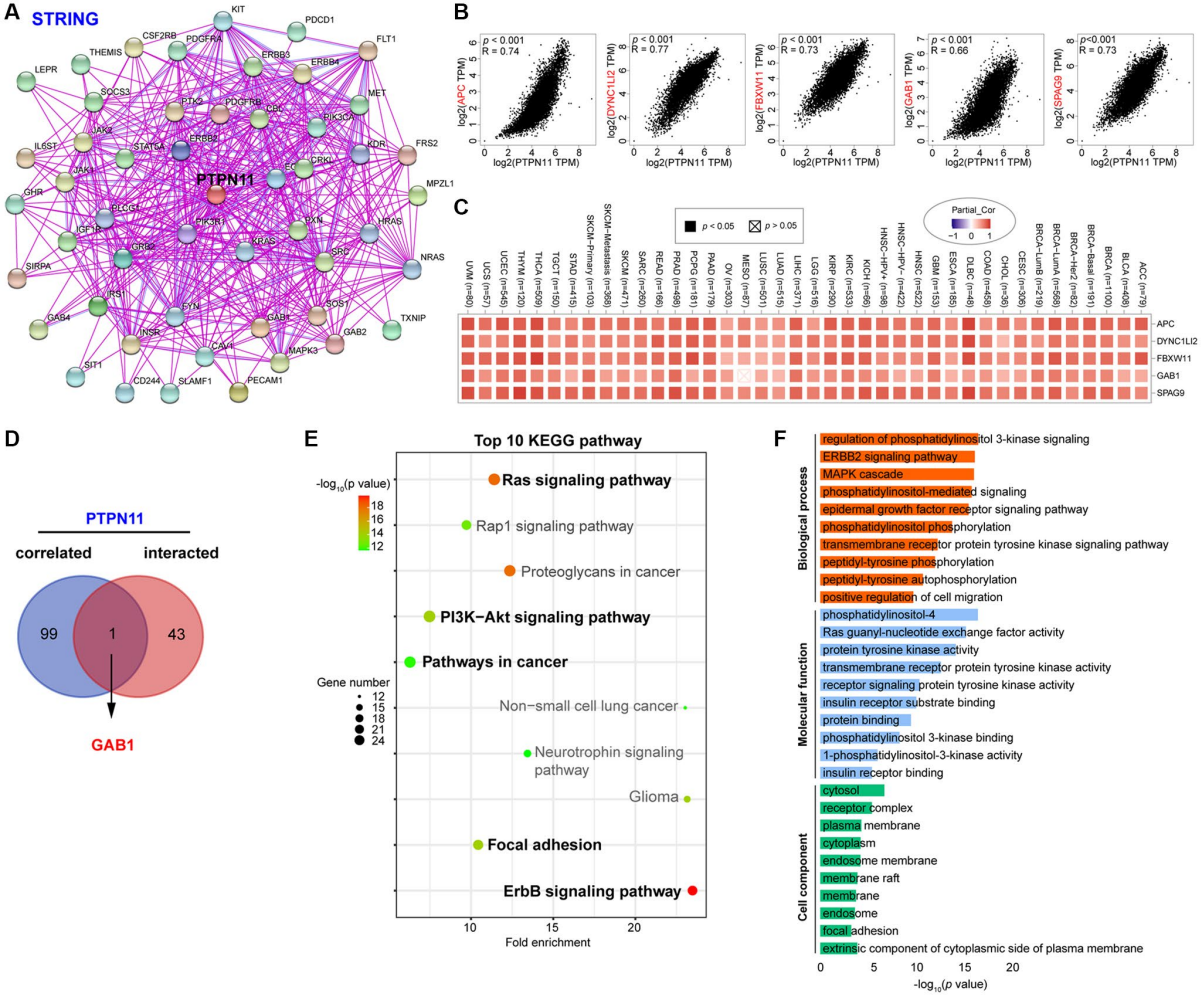
**Enrichment and pathway analysis of PTPN11-related genes.** (**A**) An experimentally validated network of interactions between PTPN11-binding proteins, as determined by STRING. (**B**) Expression association between *PTPN11* and representative genes (*APC, DYNC1LI2, FBXW11*, *GAB1*, and *SPAG9*) among the top *PTPN11*-correlated genes identified by GEPIA2. (**C**) Correlation map of *PTPN11* and *APC, DYNC1LI2, FBXW11, GAB1*, and *SPAG9* expression in TCGA cancers. (**D**) Using a Venn diagram, only GAB1 was detected in both datasets of PTPN11-binding and associated genes. (**E**) KEGG pathway evaluation of PTPN11-binding and interacted genes. (**F**) Enrichment analysis of GO terms for PTPN11-binding and interacted genes.

To study the probable molecular mechanism of PTPN11 in carcinogenesis, we compiled a network of 44 PTPN11-binding protein interactions using the STRING online database with experimental evidence (Figure 10A). Then, employing GEPIA2, we extracted the top one-hundred genes most strongly correlated with *PTPN11* expression. According to Figure 10B, the *PTPN11* expression was positively correlated with that of *APC* (APC regulator of WNT signaling pathway) (R = 0.74), *DYNC1LI2* (Dynein cytoplasmic 1 light intermediate chain 2) (R = 0.77), *FBXW11* (F-box and WD repeat domain containing 11) (R = 0.73), *GAB1* (GRB2 associated binding protein 1) (R = 0.66), and *SPAG9* (Sperm associated antigen 9) (R = 0.73) (all *p* < 0.001). In addition, the heat map confirmed the remarkably positive relation among *PTPN11* expression and above five genes in most cancers (Figure 10C). Furthermore, a Venn diagram assessment of the intersection of the 44 PTPN11-binding proteins and the 100 most highly associated genes revealed that GAB1 was the only common member (Figure 10D).

On the basis of the DAVID dataset, we also ran KEGG and Gene Ontology (GO) enriched assessments for the aforementioned two groups. The KEGG data revealed that Ras signaling pathway, ErbB signaling pathway, focal adhesion, and PI3K-Akt signaling pathway may be significantly implicated in PTPN11’s tumorigenesis effects (Figure 10E). The majority of such genes are engaged in phosphatidylinositol 3-kinase signaling in the biological process (BP) category, phosphatidylinositol-4 in the molecular function (MF) category, and cytosol in the cell component (CC) category, according to the results of the GO evaluation (Figure 10F).

## DISCUSSION

At present, tumor is still a major killer threatening human health, and its incidence is increasing year by year, which poses a great threat to human survival. Although traditional surgical resection, radiotherapy and chemotherapy have laid a solid foundation for tumor treatment, there are still some patients with malignant tumor who still progress after these treatments. According to investigations, PTPN11 is intimately associated with the beginning and advancement of some tumors. However, a thorough pan-cancer analysis of PTPN11 is still lacking. We evaluated the expression of PTPN11 in thirty-three different tumors utilizing the TCGA, GTEx, and CPTAC databases, as detailed in the current work. The results revealed that *PTPN11* expression in CHOL, COAD, DLBC, ESCA, HNSC, LGG, LIHC, STAD and THYM was significantly greater than that of corresponding healthy tissues. In BLCA, BRCA, CESC, LUAD, PAAD, and THCA, elevated *PTPN11* expression was substantially correlated with poor OS; and in CESC, PAAD, and PCPG, it was associated with poor DFS. And the degrees of *PTPN11* genetic alteration are highly linked with the outcome of some tumors. Breast cancer, clear cell RCC, HNSC, and LUAD were shown to have reduced phosphorylation levels of PTPN11. Besides, the *PTPN11* expression levels were remarkably related to the invasion of tumor-associated fibroblasts and endothelial cells, as well as TMB, MSI, and MMRs in divergent cancers. Furthermore, the enrichment analysis suggested that PTPN11 may have a crucial part in tumorigenesis via regulating Ras signaling pathway, ErbB signaling pathway, focal adhesion, and PI3K-Akt signaling pathways. Collectively, our study strongly suggests PTPN11 is a viable prognostic and therapeutic target for tumor.

Previous researches have confirmed that PTPN11 is a multifunctional non-receptor protein tyrosine phosphatase and associated with breast cancer, leukemia, lung cancer, hepatic cancer, stomach cancer as well as other cancers [11]. In accordance with prior research, our pan-cancer analysis of PTPN11 revealed differential expression in diverse tumors. *PTPN11* expression was discovered to be greater in several human cancers, notably CHOL, COAD, DLBC, ESCA, HNSC, LGG, LIHC, STAD and THYM, compared with normal tissues. Nevertheless, *PTPN11* expression was decreased in BRCA, GBM, KIRC, LUAD, PRAD, THCA, and UCEC. The discrepancies of *PTPN* expression indicated that PTPN11 played different roles in many aspects of cancer biology, including the processes of cell division, repair of DNA, metastasis, and angiogenesis. The results were consistent with previous work, which indicated that PTPN11 could either operate as an oncogenic element or a cancer inhibitor in certain illnesses [15, 25]. Besides, the Kaplan-Meier plotter data suggested the higher *PTPN11* expression was related to poorer survival prognosis in most cancers, which strongly indicates PTPN11 may represent a unique marker and therapeutic target for tumor treatment.

It is widely believed that tumorigenesis is largely influenced by gene mutations [26]. Previous work has suggested that *PTPN11* mutations were associated with genetic developmental diseases and cancers [27]. And Yang et al., confirmed that somatic *PTPN11* mutations were linked to a range of cancers, including leukemia as well as other cancers [10]. Several studies reported that gain-of function mutations of *PTPN11* are correlated with NS [28], JMML [29, 30], or colorectal cancer [31]. Moreover, Xiang et al., demonstrated that *PTPN11* mutations in cancer stem cells (CSCs) result in liver CSC expansion through activation of β-catenin signaling pathway [32]. Additionally, it is believed that *PTPN11* mutations that result in a loss of function are correlated with hypertrophic cardiomyopathy [33, 34]. In the current study, *PTPN11* mutation patterns were examined across various human cancers by cBioPortal tool and the results indicated *PTPN11* was mutated in most cancers. And the missense mutations of *PTPN11* were the most frequent DNA alterations. The correlation was then calculated between the *PTPN11* mutation status and survival prognosis of LUSC patients. The results confirmed that LUSC patients with changed *PTPN11* had a poorer OS prognostic than those without *PTPN11* modification, suggesting that PTPN11 could forecast case survival prognosis and therapy response. Moreover, the CPTAC database was utilized to evaluate the impact of PTPN11 phosphorylation on cancers. We found PTPN11 is phosphorylated at multiple sites, including S36, S562, Y546, Y584, Y542, and Y580. Notably, phosphorylation of Y542 and Y580 located at the C-terminal region are dominant targets for PTPN11 activation [35]. Phosphorylated Tyr542 binds intramolecularly to the N-SH2 domain to restore phosphatase homeostasis, whereas phosphorylated Tyr580 connects with the C-SH2 domain to enhance phosphatase activity [36].

Both cancer and stromal cells, like cancer-associated fibroblasts, endothelial cells, lymphocytes, etc., make up the TME, which is a situation favorable to tumor growth. [17, 18], which helps tumor cells escape immune surveillance. Several researches have shown PTPN11 is essential for controlling immune cell activities in the tumor environment [37]. Studies reported the SH2 domains of PTPN11 could bind to programmed cell death 1 (PD-1), an immune checkpoint target for cancer immunotherapy, to suppress T cell function and stimulate the immune escape of cancer cells [37, 38]. Considering the current study, we confirmed *PTPN11* expression had a remarkably positive relation to immune checkpoints in COAD, DLBC, LIHC, OV, PAAD, PRAD, READ, STAD, and UVM; and had a remarkably negative correlation with immune checkpoints in CESC, GBM, LUSC, SARC, SKCM, TGCT, THCA, and UCS (Supplementary Figure 2). PTPN11 inhibition has been suggested to increase the levels of intratumoral CD8^+^ T cell and tumor-associated B cell to enhance the anti-tumor immunity [39]. Additionally, tumor-associated macrophage infiltration is related to the drug resistance to immunotherapy [40]. Previous research has indicated PTPN11 could bind to the colony-stimulating factor receptor (CSF-1R) complex in response to CSF-1 stimulation in the tumor-associated macrophages to stimulate the Ras/Erk pathway, which can enhance tumor cell proliferation and migration [41]. However, the tumor immune microenvironment is very complex, and the association of *PTPN11* with immune cells and how it affects the tumor immune microenvironment remain to be clarified. It is recognized that cancer-associated fibroblasts and endothelial cells have a cancer-promoting function in the TME through the release of growth factors, cytokines, and chemokines, as well as degrading the extracellular matrix [21, 22]. A recent study also reported endothelial deletion of *PTPN11* and pharmacological inhibition could lead to tumor vascular normalization and significantly reduce the tumor growth [42]. According to the current research, how *PTPN11* expression related to the invasion of tumor-related fibroblasts and endothelial cells was investigated. The results revealed that *PTPN11* was remarkably related to the invasion levels of tumor-related fibroblasts and endothelial cells in most cancers, particularly in COAD, HNSC, LUAD, LUSC, and PAAD. Furthermore, we observed *PTPN11* was negatively related to the immunostimulants, immunosuppressants, MHCs, TILs, chemokines, and receptors in most cancers, which might further confirm the complexity of the TME. In summary, these results confirm aberrant *PTPN11* expression have a crucial part in the TME.

In the era of precision medicine, TMB can provide insights into tumor behavior and immunotherapy response. Besides, immune-checkpoint inhibitors also use MSI as a biomarker [23]. In this research, we examined the relationship between *PTPN11* expression and TMB and MSI in all TCGA cancers. A positive association between *PTPN11* expression and TMB was found in THYM, LAML, LUAD, and SKCM, while a negative association was detected in LGG and UVM. As to MSI, *PTPN11* expression was positively related to MSI in ACC, MESO, LUSC, READ, UCEC, TGCT, OV, COAD, and LUAD, while negatively related to MSI in SKCM and DLBC. There findings indicate that the *PTPN11* expression has a significant impact on TMB and MSI, and patients’ response to immune checkpoint suppression therapy. In addition, we observed *PTPN11* expression was highly correlated with MMR gene expression, which suggested that patients with related cancers may benefit from taking mutant *PTPN11* into account when assessing development and prognosis.

Furthermore, we attempted to clarify the functional properties of differentially expressed PTPN11 by integrating the PTPN11-binding proteins and *PTPN11* expression associated genes in all TCGA cancers, accompanied by KEGG pathway enriched investigation and GO enrichment analysis. The results confirmed the differentially expressed PTPN11 was mainly linked to the regulation of Ras and ErbB signaling pathway, focal adhesion, and PI3K-Akt signaling pathway. These results are in line with the previous researches. Previous studies showed that PTPN11 was commonly active in human melanoma samples and played a carcinogenic role in melanoma by regulating Ras and GSK3β signaling pathways [15]. Besides, PTPN11 could activate the Ras/Erk/MAPK signaling pathway by dephosphorylating Ras to promote cell proliferation, and activation of Ras/Erk pathway could reduce the levels of TILs, which promotes the immune escape by the tumor cells [43]. Studies also revealed that *Ptpn11* deletion in the *ErbB2* transgenic mice defends against carcinogenesis through inhibiting *ErbB2* expression [44]. Moreover, another experimental data has indicated that PTPN11 regulates the focal adhesion kinase activity through dephosphorylating pTyr397 to maintain the lamellipodia persistence to promote tumor cell migration [45]. Furthermore, PTPN11 is crucial in regulating the PI3K-Akt signaling pathway to facilitate tumor cell proliferation [27, 46]. In addition, we found *APC, DYNC1LI2, FBXW11, GAB1*, and *SPAG9* were estimated to highly associated with *PTPN11*, which showed us some evidence that PTPN11-related enrichment pathways can be used as potential biomarkers to help patients determine more precise treatment options.

In conclusion, this is the first study that shows *PTPN11* is aberrantly expressed in multiple types of cancer and clarifies how *PTPN11* expression correlates with survival of tumor patients, protein phosphorylation, TMB, MSI, MMRs, and immune cell infiltration in multiple cancers. Moreover, the present study provides a solid reference for the comprehensive features and roles of PTPN11 in tumorigenesis, which can help patients select more accurate immunotherapy regimens in the future.

## MATERIALS AND METHODS

### Assessment of PTPN11 expression in human pan-cancer

The PTPN11 expression profile in several tumors and normal tissues was explored using TIMER2’s ‘Gene DE’ package (tumor immune estimation resource, version 2) web (http://timer.cistrome.org/). The ‘Expression analysis-Box Plots’ module of the GEPIA2 (Gene Expression Profiling Interactive Analysis, version 2) web (http://gepia2.cancer-pku.cn/#analysis) was utilized for patients lacking healthy tissue samples in the TIMER2 database to examine the variation in *PTPN11* expression among different tumors and comparable normal tissues by matching TCGA and GTEx (Genotype-Tissue Expression) dataset [47]. Besides, the ‘Pathological Stage Plot’ module of GEPIA2 was chosen to investigate the *PTPN11* expression levels in distinct pathological phases.

Furthermore, the CPTAC module of UALCAN (http://ualcan.path.uab.edu/analysis.html) was used to obtain the PTPN11 protein expression levels from tumors and comparable normal tissues [48].

### Prognosis analysis

To obtain the relationship between *PTPN11* expression and survival prognosis of tumor cases, we used the Kaplan-Meier plotter (http://kmplot.com/analysis/) to investigate the OS and DFS significance map findings in 33 types of tumors. By setting "autoselect best cutoff", tumors were separated into two groups.

### Genetic alteration analysis

To clarify the genetic modification features of *PTPN11*, we made use of the ‘Cancer Types Summary’ module in cBioPortal web (https://www.cbioportal.org/). By selecting "TCGA Pan Cancer Atlas Studies" in the "Quick select" part, the modification rate, mutant type, and Copy number alteration (CNA) of PTPN11 in 33 kinds of cancers were obtained. Besides, we took advantage of the ‘Comparison’ module in cBioPortal to analyze the relationship among *PTPN11* genetic change and survival prognosis with or without *PTPN11* genetic alteration. The ‘Mutations’ module was selected to show the mutated sites in the protein structure.

### Phosphorylation analysis of PTPN11

By taking advantage of the UALCAN database, we obtained the phosphorylation degrees of PTPN11 in divergent tumors and comparable normal tissues. Besides, the PhosphoNET website (http://www.phosphonet.ca/) was used to provide a visual data of PTPN11 phosphorylation sites in primary cancer and normal tissues.

### Immunohistochemical analysis of tumor pathology

For the immunohistochemical detection of PTPN11 in different tumor tissues, we applied the HPA (https://www.proteinatlas.org) dataset to map PTPN11 protein expression across different tissues.

### Immune cell infiltration evaluation

TIMER2’s ‘Immune-Gene’ module was employed to investigate the association between *PTPN11* expression and immune cell infiltration. For the purpose of analyzing immune infiltration, we chose cancer associated fibroblasts and endothelial cells. The TIMER, CIBERSORT, CIBERSORT-ABS, QUANTISEQ, XCELL, MCPCOUNTER and TIDE algorithms were selected for estimating the immune infiltration with the purity-adjusted partial Spearman’s association test. Additionally, we investigated the association between *PTPN11* expression levels and cancer purity.

### TMB and MSI analysis

TMB is a measurable immune-response marker determined by calculating total number of gene coding mistakes, base replacements, gene insertion or removal errors per million bases [49]. DNA mismatch repair defects in tumor tissues cause MSI, which is characterized by a class of short tandem repeated DNA sequences in the genome. The presence of MSI with DNA mismatch repair defects is a clinically significant tumor marker [50]. TMB and MSI scores were calculated using mutational information from TCGA (https://tcga.xenahubs.net). And we explored the association between *PTPN11* expression and TMB as well as MSI utilizing Spearman’s method.

### Correlation analysis between *PTPN11* and immunoregulators

Using TISIDB portal (http://cis.hku.hk/TISIDB/), the association between *PTPN11* and immuno-modulators, including immunostimulants, immunosuppressants, MHC molecules, TILs, receptors, and chemokines in various tumors were analyzed.

### Investigation of PTPN11-associated gene enrichment

We were able to obtain the experimentally confirmed PTPN11-binding proteins by using STRING webpage (https://string-db.org/), with the following parameters: the required minimum interaction score was set to ‘Low confidence’, the meaning of network edges was set to ‘evidence’, the maximum number of interactors that could be displayed was set to ‘no more than 50 interactors’ and active interaction supplies to ‘experiments’. In addition, in order to determine the top 100 genes that are connected with PTPN11, the GEPIA2 ‘Similar Gene’ module was utilized. The ‘correlation analysis’ module of GEPIA2 was used to investigate the association between *PTPN11* and the aforementioned genes. Besides, the TIMER2 ‘Gene Corr’ module was utilized to collect the heatmap data of the aforementioned genes. A Venn diagram viewer (http://bioinformatics.psb.ugent.be/webtools/Venn/) was used to examine the intersect investigation of PTPN11 bound and interacted genes.

Furthermore, the DAVID website (database for annotation, visualization, and integrated discovery, https://david.ncifcrf.gov/home.jsp) was used to obtain GO enrichment assessment and KEGG pathway enriched analysis data for PTPN11 and associated genes. We then used the ‘clusterProfiler’ and ‘ggplot2’ R tools to explore and depict the enriched pathway.

### Statistical analysis

Log2 transformation was used to standardize every gene expression profiles. In order to evaluate the degree to which cancer and normal tissues differ in their expression of *PTPN11*, the Wilcox test was carried out. For the purpose of conducting a survival study on cancer patients, the Kaplan-Meier curve was utilized. In order to study the nature of the connection that exists between two variables, the partial Spearman method was applied. For the purpose of processing all of the statistical analyses, R software, version 4.0.2, was used. *P* less than 0.05 was deemed statistical significance.

## Supplementary Materials

Supplementary Figures
