# Supplementary material for: A pan-cancer analysis confirms PTPN11’s potential as a prognostic and immunological biomarker
Source: Aging (Albany NY). 2022 Jul 8;14(13):5590–610. doi: 10.18632/aging.204171 (PMC9320542; doi:10.18632/aging.204171)
Supplement: Supplementary Figures [file aging-14-204171-s001.pdf]

SUPPLEMENTARY FIGURES

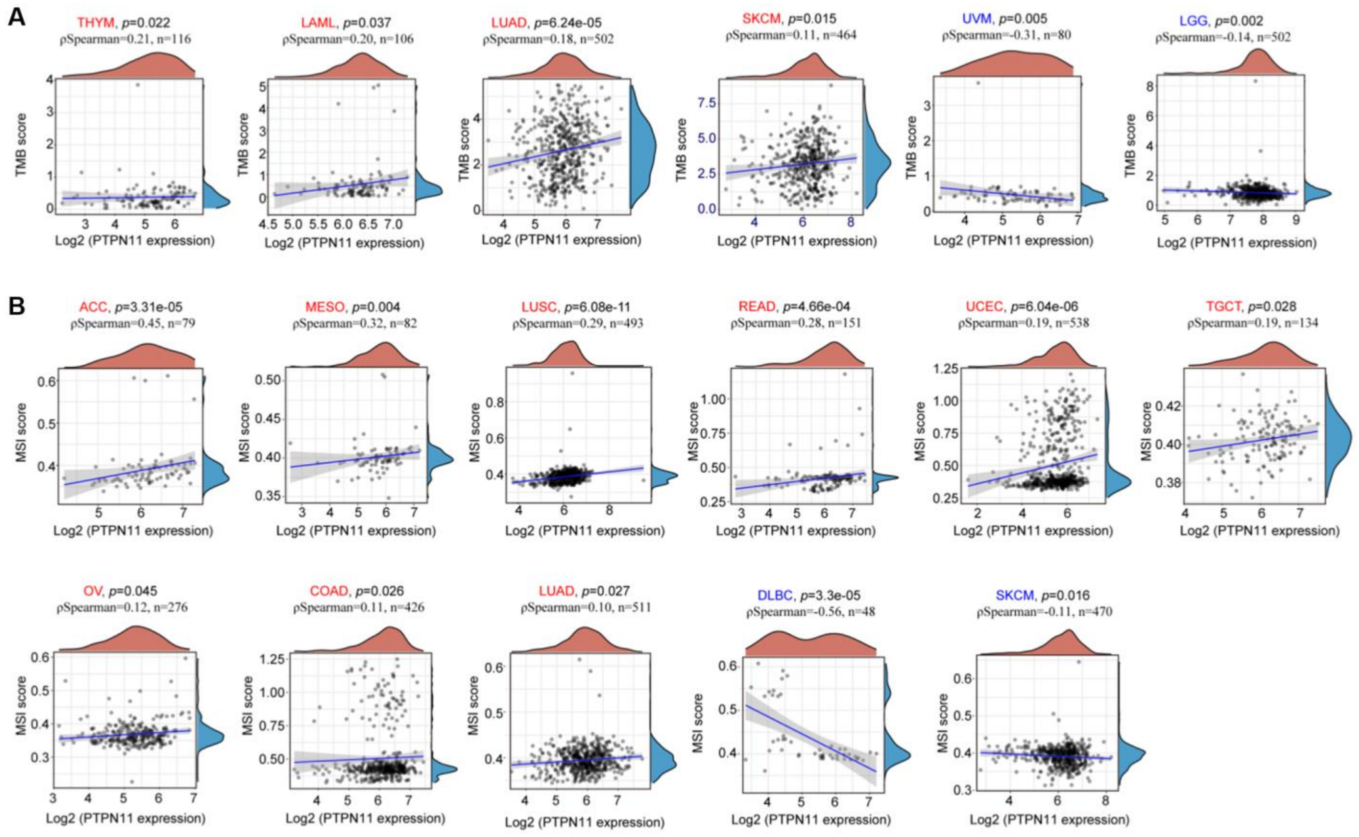

Supplementary Figure 1. Correlation analysis between the *PTPN11* gene expression and TMB and MSI in human pan-cancer. (**A**) Relationship between the *PTPN11* gene expression and TMB in THYM, LAML, LUAD, SKCM, UVM, and LGG. (**B**) Relationship between the *PTPN11* gene expression and MSI in ACC, MESO, LUSC, READ, UCEC, TGCT, OV, COAD, LUAD, DLBC, and SKCM. Correlation analysis was conducted by Spearman's method.

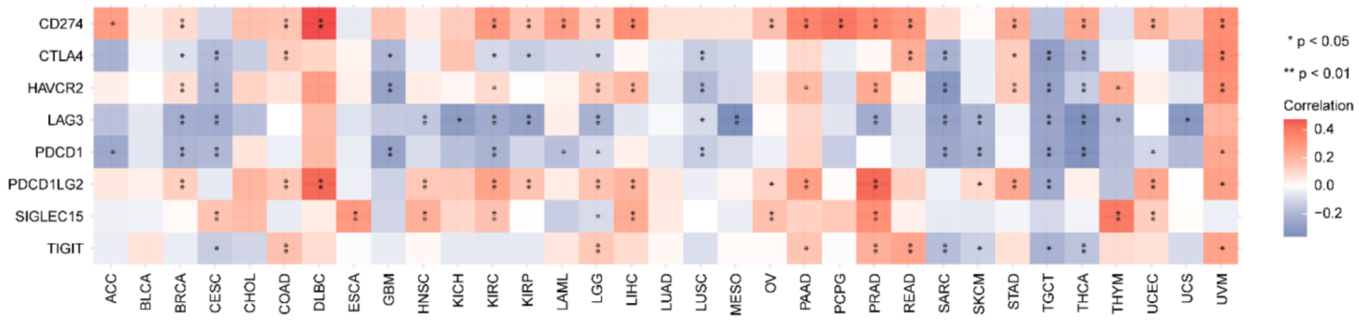

Supplementary Figure 2. Correlation analysis of *PTPN11* expression with common immune checkpoints in human pan-cancer.

Abbreviations

TCGA cancer abbreviations

ACC: Adrenocortical carcinoma; BLCA: Bladder Urothelial Carcinoma; BRCA: Breast invasive carcinoma; CESC: Cervical squamous cell carcinoma and endocervical adenocarcinoma; CHOL: Cholangiocarcinoma; COAD: Colon adenocarcinoma; DLBC: Lymphoid Neoplasm Diffuse Large B-cell Lymphoma; ESCA: Esophageal carcinoma; GBM: Glioblastoma multiforme; HNSC: Head and Neck squamous cell carcinoma; KICH: Kidney Chromophobe; KIRC: Kidney renal clear cell carcinoma; KIRP: Kidney renal papillary cell carcinoma; LAML: Acute Myeloid Leukemia; LGG: Lower Grade Glioma; LIHC: Liver hepatocellular carcinoma; LUAD: Lung adenocarcinoma; LUSC: Lung squamous cell carcinoma; MESO: Mesothelioma; OV: Ovarian serous cystadenocarcinoma; PAAD: Pancreatic adenocarcinoma; PCPG: Pheochromocytoma and Paraganglioma; PRAD: Prostate adenocarcinoma; READ: Rectum adenocarcinoma; SARC: Sarcoma; SKCM: Skin Cutaneous Melanoma; STAD: Stomach adenocarcinoma; TGCT: Testicular Germ Cell Tumor; THCA: Thyroid carcinoma; THYM: Thymoma; UCEC: Uterine Corpus Endometrial Carcinoma; UCS: Uterine Carcinosarcoma; UVM: Uveal Melanoma.
